# Aspect clinico-électrocardiographique d'embolie pulmonaire masquant une dissection aortique révélé par l'angioscanner thoracique

**DOI:** 10.11604/pamj.2017.28.3.12605

**Published:** 2017-09-04

**Authors:** Daniella Rakotoniaina Masinarivo, Jenny Larissa Rakotomanana

**Affiliations:** 1 Service USIC, CHU Joseph Raseta Befelatanana, Antananarivo, Madagascar

**Keywords:** Douleur thoracique, embolie pulmonaire, dissection aortique, angio-scanner thoracique, Chest pain, pulmonary embolism, aortic dissection, thoracic CT angiography

## Abstract

We here report the case of a 52-year old hypertensive, obese woman (BMI 32,46 kg/m^2^) with a past history of smoking and without evidence-based risk factors of venous thromboembolism, hospitalized for left chest pain radiating to the dorsolumbar region associated with dyspnoea. Clinical examination on hopitalization showed left blood pressure 100/60 mmHg, tachycardia 100/min, oxygen desaturation index at 88% with the patient breathing ambient air, normal cardiopulmonary auscultation, peripheral pulses palpable and no symptoms of phlebitis of the lower limbs. The ECG showed right axis deviation, S_1_Q_3_ pattern, right ventricular hypertrophy and right bundle branch block (A, B, C). The patient underwent emergency thoracic CT angiography objectifying aortic dissection from the origin of the aorta to the iliac bifurcation (Stanford A). Our patient received medical care based on blood pressure and heart rate control as well as on analgesics, with good evolution in the absence of surgical means.

## Image en médecine

Il s'agit d'une femme de 52 ans, hypertendue, tabagique et obèse (IMC à 32,46 kg/m^2^), sans facteurs de risques thromboembolique évident, hospitalisée pour une douleur thoracique gauche à irradiation dorso-lombaire associée à une dyspnée. L'examen clinique à l'entré objectivait une pression artérielle gauche à 100/60 mmHg, une tachycardie à 100/min, une désaturation en oxygène à 88% à l'air ambiant, avec auscultation cardio-pulmonaire sans particularité, pouls périphériques perçus et absence de signes de phlébite des membres inférieurs. L'ECG montrait un axe droit, un aspect S_1_Q_3_, une hypertrophie ventriculaire droite et un bloc de branche complet droit (A, B, C). L'angioscanner thoracique effectué en urgence objectivait une dissection aortique allant de l'origine de l'aorte jusqu'à la bifurcation iliaque soit de type Stanford A. Notre patiente a été traité médicalement par contrôle de la pression artérielle et de la fréquence cardiaque ainsi que des antalgiques, avec une bonne évolution devant l'absence de moyen chirurgical.

**Figure 1:**
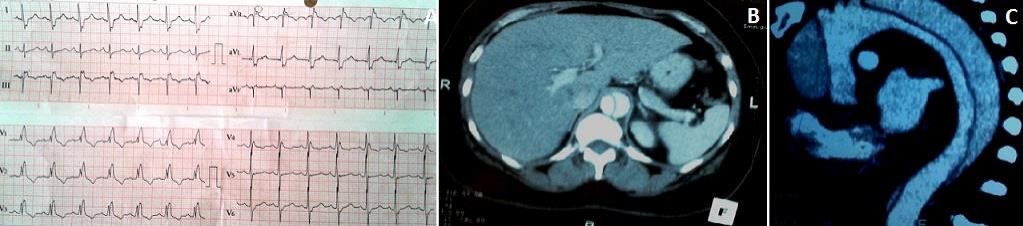
(A) ECG bloc de branche droit avec aspect S_1_Q_3_; (B) angioscanner thoracique coupe transversale: image de dissection aortique, aspect en "balle de ping-pong"; (C) angioscanner thoracique: coupe longitudinale: image de dissection aortique, avec le faux et le vrai chenal

